# HPLC Method Development for Quantification of Doxorubicin in Cell Culture and Placental Perfusion Media

**DOI:** 10.3390/separations5010009

**Published:** 2018-01-24

**Authors:** Mansi Shah, Luke Bourner, Shariq Ali, Sanaalarab Al-Enazy, Menatallah M. Youssef, Morgan Fisler, Erik Rytting

**Affiliations:** 1 Department of Obstetrics & Gynecology, University of Texas Medical Branch, 301 University Boulevard, Galveston, TX 77555-1062, USA; 2 Department of Pharmacology and Toxicology, University of Texas Medical Branch, Galveston, TX 77555-1062, USA; 3 School of Medicine, University of Texas Medical Branch, Galveston, TX 77555-1062, USA; 4 Department of Pharmaceutical Analytical Chemistry, Ain-Shams University, Cairo 1156, Egypt; 5 Department of Obstetrics & Gynecology, Medical University of South Carolina, Charleston, SC 29425, USA

**Keywords:** doxorubicin, BeWo cell culture media, placenta, high performance liquid chromatography, fluorescence, anthracycline, analytical method, perfusion

## Abstract

Assessment of drug transport across the placenta is important in understanding the effect of drugs on placental and fetal health. These phenomena can be studied in both in vitro cell lines and ex vivo placental perfusions. We have successfully developed a sensitive yet simple high performance liquid chromatography (HPLC) method coupled with fluorescence detection to determine the concentration of doxorubicin (DXR) in cell culture media for transport studies in human trophoblast cells (BeWo, b30 clone) and in fetal media for placental perfusion experiments. The method was developed based on a protein precipitation technique and was validated in both media types for linearity, intra-day, and inter-day precision and accuracy. The relationship of peak area to concentration was linear with *R*^2^ values of 0.99 or greater obtained over the concentration range of 1.5 to 15,000 ng/mL. Despite the high concentrations of albumin in fetal perfusion media (30 mg/mL), the lower limits of detection and quantification for DXR were found to be 1.5 and 5 ng/mL, respectively. This analytical method may be used to study the transport of DXR across BeWo cells and human placenta during placental perfusion studies.

## 1. Introduction

The occurrence of breast cancer in pregnant women has increased over the past few years, complicating approximately 1 in 3000 pregnancies. Moreover, treatment strategies involving chemotherapy can be toxic to the fetus [1]. Pregnancy is a dynamic state in which the body undergoes several anatomical and physiological changes. These changes can alter the pharmacokinetics and pharmacodynamics of the drug compared to non-pregnant subjects [2]. However, pharmacokinetic studies have rarely been performed on pregnant women receiving chemotherapy. Doxorubicin is commonly prescribed during pregnancy for the treatment of malignancies such as breast cancer, ovarian cancer, and solid tumors [3], despite the fact that the FDA has defined it as pregnancy category D (positive evidence of risk). Therefore, it is imperative to study the transplacental transfer of the drug itself and its liposomal formulation (Doxil^®^) and understand its implications on fetal development.

Doxorubicin (DXR) is an anthracycline antibiotic isolated from the bacterium *Streptomyces peucetius* var. *caesius* and is available as a hydrochloride salt. Doxorubicin has multiple mechanisms of action, including DNA intercalation and prevention of transcription, inhibition of topoisomerase II, and induction of cytotoxicity by the formation of oxygen free radicals [4]. DXR is known to be rapidly and extensively distributed to various organs such as the heart, liver, kidney, and spleen, but little is known about its transport and accumulation in the placenta [4–6]. Therefore, to investigate the mechanisms involved in the transplacental transfer of doxorubicin, various in vitro and ex vivo models can be studied [7]. The BeWo (b30 clone) cell line, which is a model of the human placental trophoblast, and the dually perfused human placental lobule are both widely used in vitro and ex vivo models to study the transplacental transfer of various drugs and their metabolites [7,8]. In order to study the transplacental transport of DXR across BeWo cells and placenta during placental perfusion studies, it is necessary to have simple, selective, and validated high performance liquid chromatography (HPLC) methods for determining DXR concentrations in media.

A handful of analytical methods for the determination of doxorubicin in biological fluids are available; however, to the best of our knowledge, none have been published for quantification in DMEM/F12 cell culture media and/or placental perfusion media. Some of the methods involve sample preparations such as liquid-liquid extraction [9–12] or solid phase extraction [13], which can be tedious and require the use of an internal standard. Other methods with protein precipitation involved the use of perchloric acid [14], which can cause deterioration of both the analyte and typical silica-based reversed-phase HPLC columns. While screening materials to use as a protein precipitant, we observed instability in DXR in the presence of perchloric acid. Other methods involve mass spectrometry, which may be highly sensitive but cost-prohibitive for many laboratories [15]. If the drug of interest is inherently fluorescent, fluorescence detection is often preferable because it is simple, inexpensive, and more sensitive in comparison to ultraviolet absorption. A recent method based upon protein precipitation was used to determine doxorubicin in arthritic joints, but it has a lower limit of quantification of only 0.8 μg/mL [16]. Therefore, even though several methods have been used to detect DXR in biological fluids, there is great benefit to developing a simple method with a low limit of quantification (5 ng/mL) for the assessment of DXR in cell culture and placental perfusion media.

For these reasons, we have developed a sensitive reversed-phase HPLC method using fluorescence detection for the quantification of DXR in DMEM/F12 cell culture medium and M199 placental perfusion medium containing 30 mg/mL human serum albumin. Since DXR exhibits high protein binding (45–85%), it is very important to use the physiological concentration of albumin, which can affect the transplacental transport of DXR [17,18]. Method development in placental perfusion medium is particularly challenging due to this high concentration of albumin. The method developed was reliable and highly sensitive. We believe that the simplicity of the sample preparation method and the portability between the two different types of media makes this an accessible and valuable method for laboratories studying doxorubicin’s interactions with the placenta.

## 2. Materials and Methods

### 2.1. Chemicals and Reagents

Doxorubicin hydrochloride was obtained from Oakwood Chemical (Estill, SC, USA). Cell culture medium for the BeWo b30 cell line contains Dulbecco’s Modified Eagle’s Medium/Ham’s F-12 50/50 mixture without phenol red (Mediatech, Manassas, VA, USA) with 10% fetal bovine serum (Atlanta Biologicals, Flowery Branch, GA, USA), antibiotic/antimycotic containing 10,000 units/mL penicillin, 10,000 μg/mL streptomycin, and 25 μg/mL amphotericin B (Gibco^®^, Carlsbad, CA, USA), 100X MEM nonessential amino acid solution (Sigma, St. Louis, MO, USA), and 200 mM L-glutamine (Mediatech, Manassas, VA, USA). Placental perfusion medium was prepared using dextran (Sigma Aldrich, St. Louis, MO, USA), human serum albumin fraction V (Calbiochem, La Jolla, CA, USA), and medium 199 (Sigma Aldrich). Acetonitrile of HPLC grade, acetone, monobasic potassium phosphate, and phosphoric acid were obtained from Fisher Scientific (Fair Lawn, NJ, USA). Deionized water was obtained from a Millipore Synergy UV Ultrapure Water System (Millipore, Billerica, MA, USA).

### 2.2. Chromatographic Instruments and Conditions

The HPLC system (Waters, Milford, MA, USA) was comprised of a Waters 1525 Binary HPLC pump, a Waters 2707 Autosampler, and a Waters 2475 multi λ fluorescence detector. For both methods, a C-18 column (150 mm × 4.6 mm, 5 μm particle size, and 100 Å pore size, AkzoNobel/Kromasil, Brewster, NY, USA) was used at ambient temperature.

The mobile phase consisted of an aqueous phase (A) containing 20 mM KH_2_PO_4_ and 0.1% (*v*/*v*) phosphoric acid and an organic phase (B), which was acetonitrile. Flow was set at 1.0 mL/min. A gradient method was used in which the mobile phase started as 75% A and 25% B and changed in a linear manner to 60% A and 40% B by 7 min. Between 7 and 10 min, the mobile phase ratio was held constant. The mobile phase then reverted to 75% A and 25% B by 12 min and was held constant to baseline until 15 min. A wash vial containing initial mobile phase was injected between samples to avoid sample carryover. Peaks were monitored using fluorescence detection at an excitation wavelength of 480 nm and an emission wavelength of 550 nm using the fluorescence detector.

### 2.3. Sample Preparation

Cell culture medium for the analytical method validation of DXR was prepared as per the protocol in which BeWo b30 cells are typically grown [7]. In brief, cell culture media containing Dulbecco’s Modified Eagle’s Medium/Ham’s F-12 50/50 mixture without phenol red (Mediatech, Manassas, VA, USA) containing 10% fetal bovine serum (Atlanta Biologicals, Flowery Branch, GA, USA), antibiotic/antimycotic containing 10,000 units/mL penicillin, 10,000 μg/mL streptomycin, and 25 μg/mL amphotericin B (Gibco^®^, Life Technologies, Carlsbad, CA, USA), 100X MEM nonessential amino acid solution (Sigma, St. Louis, MO, USA) and 200 mM L-glutamine (Mediatech, Manassas, VA, USA) was prepared and stored at 4 °C until further use.

For the analytical method validation of DXR in placental perfusion medium, fetal perfusion media was used. Here, we have selected only fetal perfusion medium as the only difference between maternal and fetal perfusion media is the level of dextran. Maternal perfusion medium contains 3.0 mg/mL, whereas fetal perfusion medium contains 12.0 mg/mL dextran. Fetal perfusion medium was prepared using deionized water in which solid medium 199 with Earle’s salts (11 mg/mL), dextran (12 mg/mL), heparin (29 μg/mL), gentamicin (33.3 μg/mL), sulfamethoxazole (80 μg/mL), trimethoprim (16 μg/mL), and human serum albumin (30 mg/mL) were dissolved and the pH of the media was adjusted to 7.4 using sodium bicarbonate [18]. All of the above mentioned chemicals used in fetal perfusion media were obtained from Sigma Aldrich (St. Louis, MO, USA). The prepared perfusion medium was stored at 4 °C and was used within a week.

A stock solution of 300 μg/mL of doxorubicin hydrochloride was prepared in purified water. Using this stock solution, 15 μg/mL DXR hydrochloride in cell culture media (DMEM/F12, phenol red free) was prepared. The series of dilutions were prepared in cell culture media. Thereafter, in order to remove the proteins, samples for HPLC analysis were prepared by a protein precipitation technique. Briefly, in a microcentrifuge tube, 250 μL of media samples with known concentrations of doxorubicin were added to 1.0 mL of acetone (1:4 ratio). Samples were then centrifuged at 16,100× *g* for 20 min to pellet the precipitated proteins. At the end of the centrifugation, 1.0 mL of supernatant was collected in a glass vial and allowed to evaporate in a vacuum oven (Napco Model No. 5831, Napco, Tualatin, OR, USA) using a chemical resistant pump (Labnet, Model No. D4000, Labnet, Edison, NJ, USA) for 30 min, followed by drying under a nitrogen stream (ZipVap 24, Glas-Col, Terre Haute, IN, USA) until complete dryness. Once dried, 100 μL of initial mobile phase (75% aqueous phase and 25% organic phase) was used to reconstitute the residual content, and the sample was transferred to an HPLC vial containing an insert, and 50 μL was injected into the HPLC system using partial loop with needle overfill.

### 2.4. Transplacental Transfer of DXR across BeWo Cells and the Dually Perfused Human Placental Lobule

To further confirm the validity and suitability of our validated HPLC method, a short experiment in the presence of BeWo cells and a placental perfusion of DXR was performed. For transport across the BeWo cells, a method was followed as per a previous report [19], and sample was collected from the basolateral chamber at 30 min. In the case of placental perfusion, a published method was followed with slight modification [20]. In brief, a placenta from an uncomplicated pregnancy at the John Sealy Hospital Labor and Delivery unit was immediately collected. After thorough evaluation, one placental lobule was selected, and a fetal artery and vein were catheterized. The maternal side of the placenta was perfused at a flow rate of 12.0 mL/min, whereas the fetal side was perfused at 3 mL/min. After addition of doxorubicin into the material reservoir, 1.0 mL of sample was withdrawn at 30 min from the fetal reservoir and analyzed by HPLC for doxorubicin determination to check the suitability of the HPLC method. Care was taken to supply 95% nitrogen and 5% carbon dioxide to the fetal reservoir and 95% oxygen and 5% carbon dioxide to the maternal reservoir (250 mL) to ensure acceptable oxygen transfer and placental viability.

### 2.5. Assay Validation

#### 2.5.1. Linearity, Precision, and Accuracy

The calibration curve was prepared using the peak areas obtained from the chromatograms. Linear regressions were made using SigmaPlot (version 13.0) based on 1/*y* weighting to minimize the sum of squares of relative errors across the concentration range (1.5–15,000 ng/mL). In order to calculate various parameters including intra-day precision, inter-day precision, and accuracy, ICH guidance for method validation of analytical procedures was followed [21]. Intra-day precision was calculated based upon relative standard deviation (%RSD) of the peak area from three concentrations (6 determinations per concentration) prepared on the same day and inter-day precision was calculated as the %RSD of peak areas of three concentrations (*n* = 6 determinations per concentration per day) over three days (*n* = 18). Accuracy was determined as the average peak area for three concentrations (*n* = 6) as a percent of the value for their corresponding concentrations based on the linear regression calculation. The matrix effect of doxorubicin was investigated in six batches of each type of media with low and high concentrations undergoing the same centrifugation, drying, and reconstitution steps. The matrix factor (MF) was calculated as MF = (analyte peak area of the matrix-matched calibration standard)/(analyte peak area of a pure standard sample) [22]. The variation of the matrix factor (relative standard deviation) was <15%.

#### 2.5.2. Limits of Detection and Quantification

The peak area from the blank samples at the same retention time of the DXR was analyzed to determine the noise. The lower limit of detection (LLOD) and lower limit of quantification (LLOQ) were based on the concentration at which the peak area of the drug was at least three times and ten times higher than the noise (blank samples) at that retention time, respectively.

#### 2.5.3. Stability of Samples

Stability of the samples in each of the media were studied for 1 week at 4 °C. These conditions were chosen based on the temperature and maximum storage time to which typical experimental samples are subjected. Results were calculated as a percent change. Percent change was calculated by the difference obtained in the peak area of the same sample on Day 1 and on Day 8, divided by the peak area of that sample on Day 1.

## 3. Results and Discussion

### 3.1. HPLC Method Development

**Figure 1:**
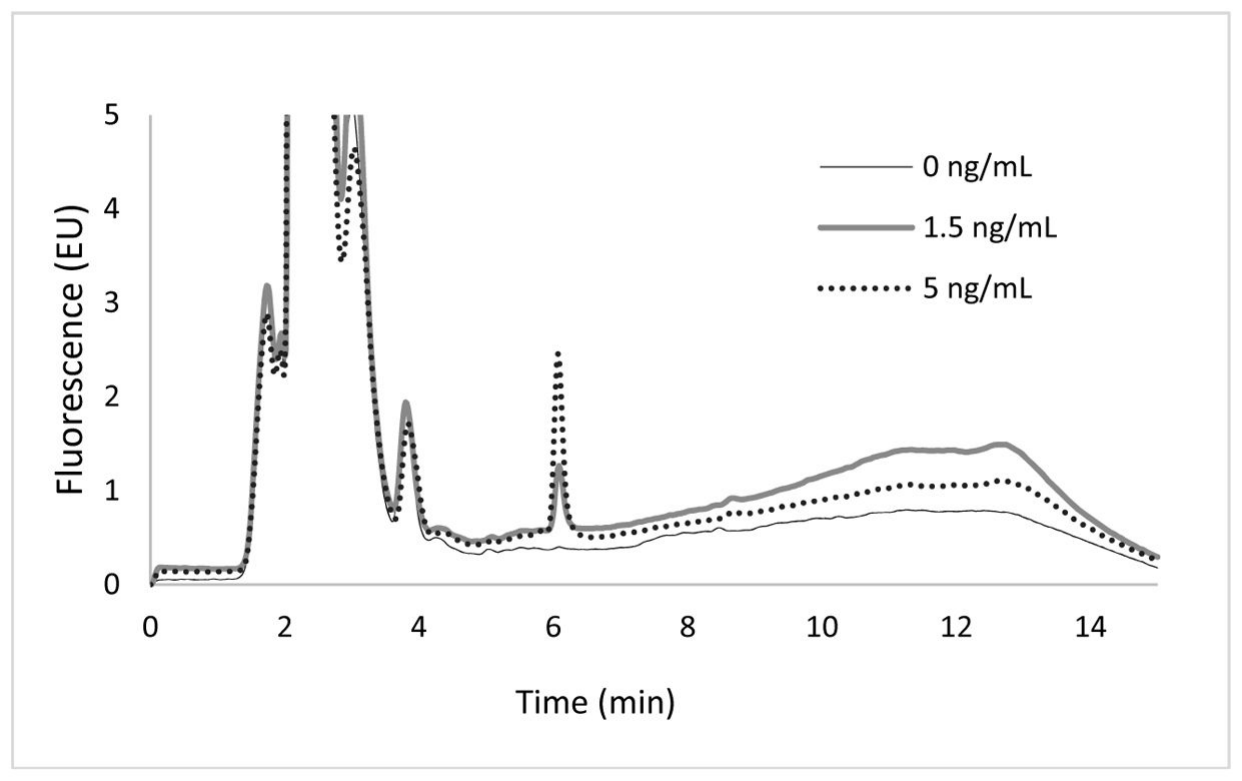
Chromatograms showing a peak for doxorubicin in DMEM/F12 medium analyzed with a fluorescence detector for a blank (0 ng/mL), a lower limit of detection (1.5 ng/mL), and a lower limit of quantification (5 ng/mL) at a retention time of 6.0 ± 0.1 min.

**Figure 2:**
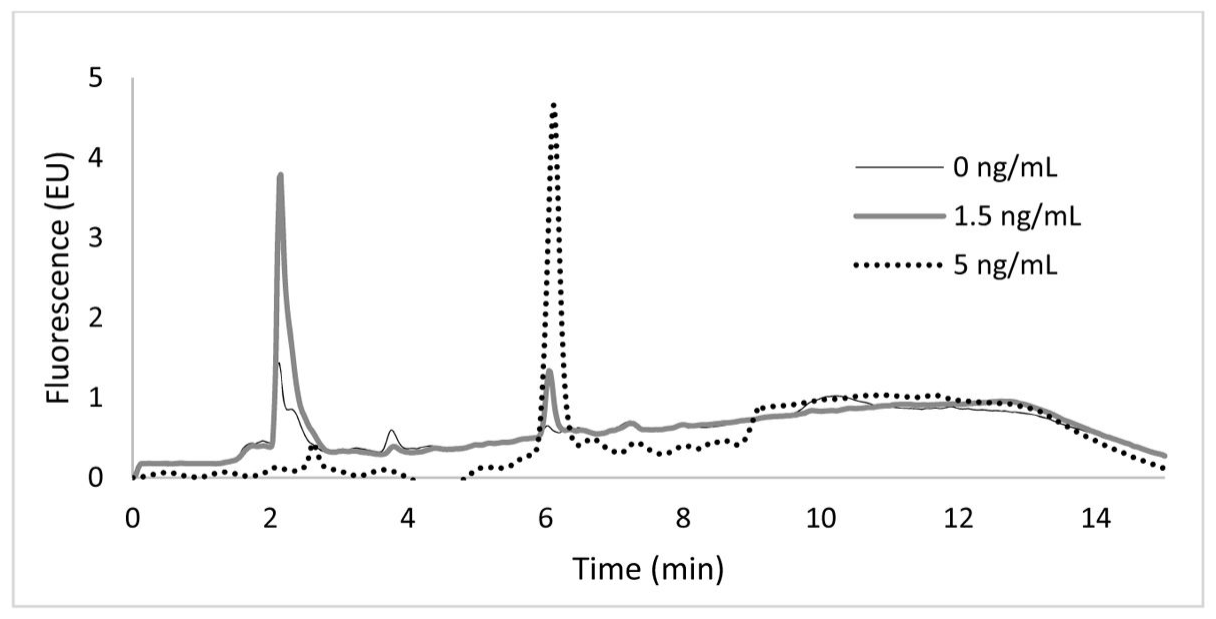
Chromatograms showing a peak for doxorubicin in fetal perfusion medium analyzed with a fluorescence detector for blank (0 ng/mL), lower limit of detection (1.5 ng/mL), and lower limit of quantification (5 ng/mL) samples at a retention time of 6.0 ± 0.1 min.

Due to the intrinsic fluorescence of doxorubicin, concentrations were analyzed using a fluorescence detector (λ_ex_: 480 nm; λ_em_: 550 nm). Samples were analyzed in both DMEM/F12 media and fetal perfusion media. Irrespective of the media used, the DXR peak was at a retention time of 6.0 ± 0.1 min with no interfering peaks (Figures 1 and 2). In addition, low and consistent noise obtained following the sample treatment confirmed the reproducibility and absence of contamination from samples even after the solvent evaporation step.

### 3.2. Linearity, Precision, and Accuracy

The relationship of peak area to concentration was found to be linear throughout the concentration range studied for both media. Using SigmaPlot, linear regressions were generated based on 1/*Y* weighting and the results of slope, intercept, and correlation coefficient are shown in Table 1. A correlation coefficient of 0.99 or greater was obtained for the concentration range (1.5–15,000 ng/mL) for all three days with both types of media. The data calculated for intra-day precision, inter-day precision, and accuracy are shown in Table 2. The intra-day precision and inter-day precision were found to be less than 15% for all three studied concentrations in DMEM/F12 and placental perfusion media, which was within acceptable limits. Accuracy was also found to be between 90% and 110% for all the concentrations studied. As shown in Table 3, the matrix factor of doxorubicin is in the range of 97–113% with a variation <11%. These results indicate that no significant matrix effect was observed in either type of media.

### 3.3. Limits of Detection and Quantification

Using the protein precipitation technique, the lower limit of detection (LLOD) was found to be 1.5 ng/mL in both types of media. This was of particular concern because the albumin concentration in perfusion media (30 mg/mL) is much higher than that in cell culture media (22 mg/mL), which could have led to differences in LLOD at low concentrations. Even though the precision at the LLOD was found to be poor due to background noise interference at this very low concentration, this analytical method still had a limit of quantification of 5 ng/mL in DMEM/F12 and placental perfusion media with acceptable accuracy and precision. This LLOQ is comparable to that of methods requiring liquid-liquid extraction [23].

### 3.4. Sample Stability

Doxorubicin was found to be stable for 1 week at 4 °C in DMEM/F12 as well as in placental perfusion media. Peak areas of doxorubicin in both types of media at all concentrations tested were comparable with minimal change following storage (Table 4). No additional peaks or alterations in retention time were observed. These results indicate that the storage conditions chosen are suitable for experimental samples.

### 3.5. Transplacental Transfer of DXR across BeWo Cells and the Dually Perfused Human Placental Lobule

**Figure 3:**
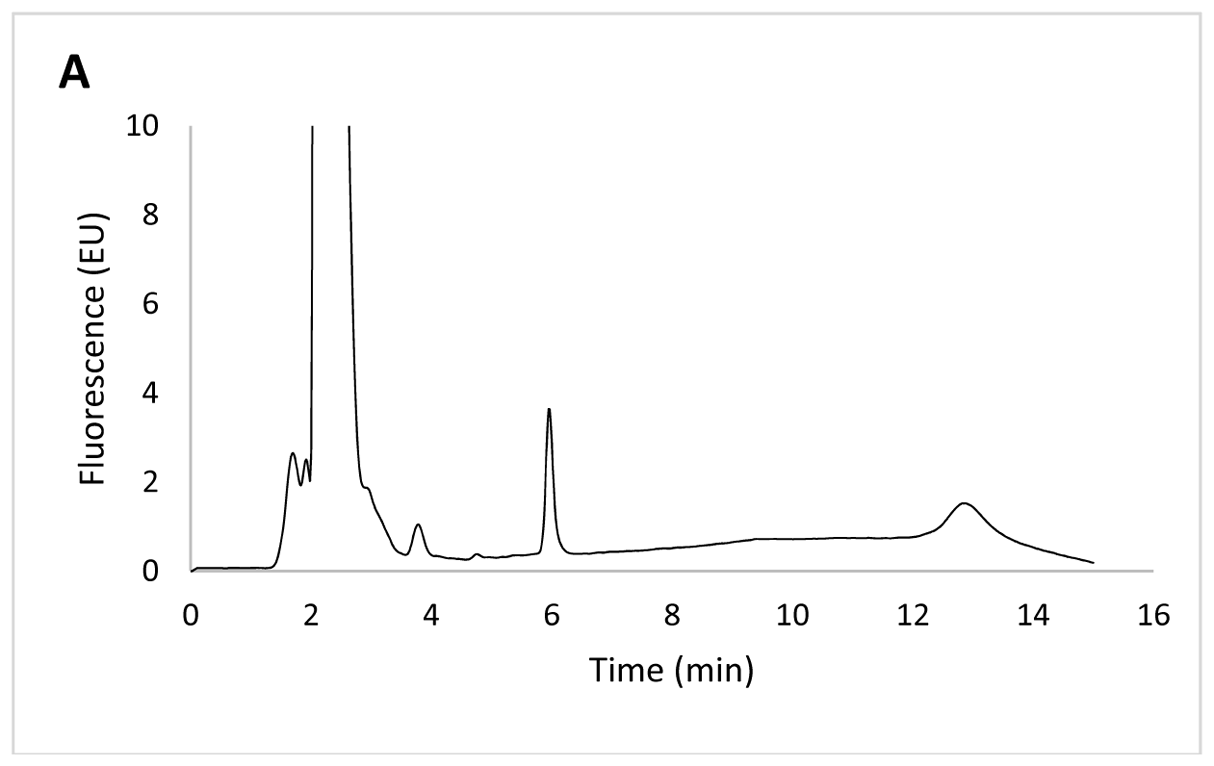

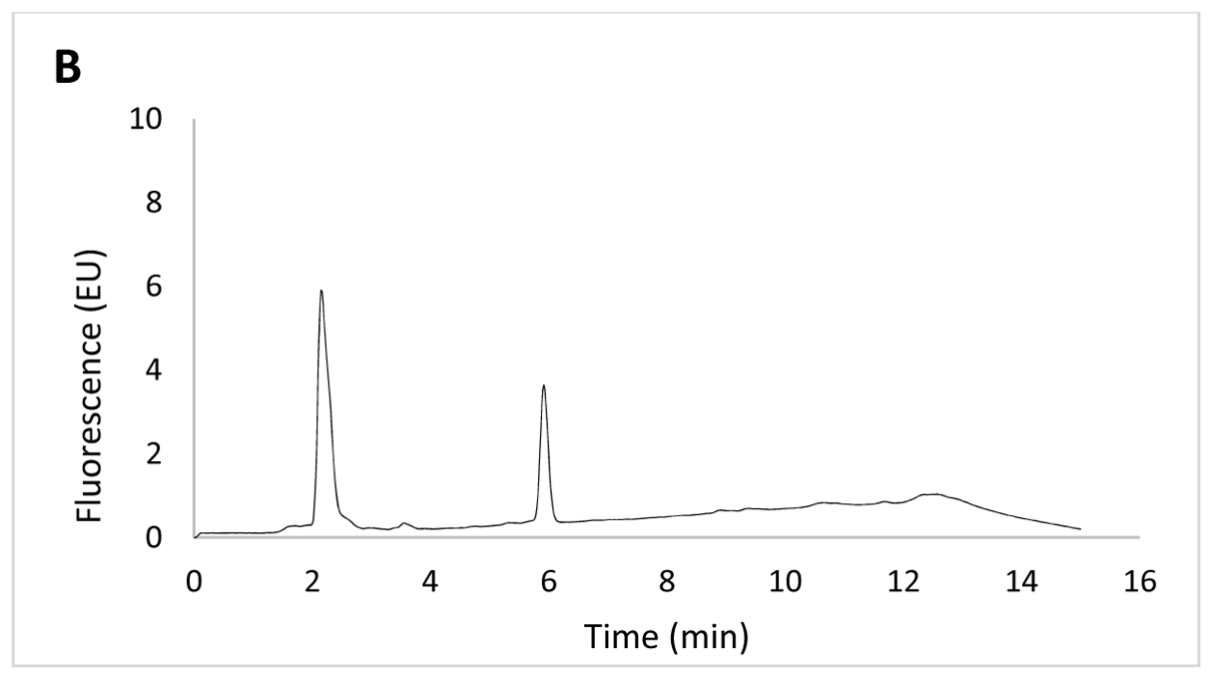
Chromatograms showing a peak for doxorubicin (retention time of 6 min) from samples collected from a transport study in BeWo cells (**A**) and placental perfusion (**B**) analyzed with the developed HPLC method.

The samples collected after the transport study and placental perfusion were analyzed using the developed HPLC method. Representative chromatograms are shown in Figure 3. It appears that the obtained chromatograms show DXR eluting at the same retention time, irrespective of whether the drug was studied using a BeWo cell monolayer or a placental lobule. Despite the fact that the placental lobule contains blood and other substances, the protein precipitation technique applied herein has successfully allowed the detection of DXR without any interference.

## 4. Conclusions

The analytical method developed in this study was found to be simple and sensitive in both types of media tested. Despite the high albumin concentration used in fetal perfusion media along with other components, the method was able to resolve, identify, and quantify DXR without substantial interference from matrix components. Excellent linearity, precision, and accuracy across all the concentrations studied were obtained using this technique. This method involving protein precipitation and fluorescence detection had lower limits of detection and quantification of 1.5 ng/mL and 5.0 ng/mL, respectively, while bypassing the need for more expensive instrumentation such as LC/MS. Therefore, this method can be used further with great confidence to understand the transplacental transfer of doxorubicin across BeWo cells and in placental perfusion experiments.

## Figures and Tables

**Table 1:** Linear regression characteristics.

Regression Parameters	Day 1	Day 2	Day 3	Average	Standard Deviation	RSD (%)
**DMEM/F-12**						

**Intercept**	4191.9	2513.3	3749.7	3485.0	870.1	24.9
**Slope**	3.761 × 10^7^	3.940 × 10^7^	3.753 × 10^7^	3.818 × 10^7^	1.06 × 10^6^	2.7
***R*^2^**	0.9996	0.9988	0.9954	0.9979	0.002	0.2

**Fetal M199**						

**Intercept**	10253.5	9697.6	10453.9	10135.0	391.9	3.9%
**Slope**	4.592 × 10^7^	4.341 × 10^7^	4.228 × 10^7^	4.387 × 10^7^	1.86 × 10^6^	4.2%
***R*^2^**	0.9974	0.9966	0.9993	0.9978	0.001	0.1%

**Table 2:** Precision and accuracy.

Concentration (μg/mL)	Intra-Day Precision (%)			Inter-Day Precision (%)	Accuracy (%)

	Day 1	Day 2	Day 3		
**DMEM/F-12**					

0.05	5.7	1.2	1.9	7	96.4
5	2.6	5.3	9.1	3.3	101.6
10	2.7	10.3	6.4	1.2	100.1

**Fetal M199**					

0.05	4.1	3.5	3.6	11.3	103.1
5	2.8	2.8	3.4	0.9	100.2
10	0.5	1.6	3.2	6.3	102.8

**Table 3:** Matrix factor of doxorubicin in DMEM/F12 and fetal M199 media (*n* = 6).

Measured Concentration (μg/mL)	Matrix Factor (%)	

	DMEM/F12	Fetal M199
0.01	113.1 ± 6.4	101.1 ± 10.8
10.0	109.7 ± 7.0	96.6 ± 9.4

**Table 4:** Stability of samples at 4 °C for 1 week.

Nominal Concentration (μg/mL)	Measured Concentration (μg/mL)		

	Day 1	Day 8	% Change
**In DMEM/F12 cell culture media**			

0.05	0.050	0.049	1.3%
5	5.02	4.44	11.5%
10	10.19	9.57	6.1%

**In fetal placental perfusion media**			

0.05	0.052	0.048	8.5%
5	4.80	4.45	7.2%
10	10.50	10.53	0.3%
